# Associations Between Endometriosis and Gut Microbiota

**DOI:** 10.1007/s43032-021-00506-5

**Published:** 2021-03-03

**Authors:** Agnes Svensson, Louise Brunkwall, Bodil Roth, Marju Orho-Melander, Bodil Ohlsson

**Affiliations:** 1 Department of Internal Medicine, Skåne University Hospital, Lund University, Jan Waldenströms street 15, floor 5, 20502 Malmö, Sweden; 2 Department of Clinical Sciences in Malmö, Lund University, Malmö, Sweden

**Keywords:** Endometriosis, Gastrointestinal symptoms, Gut microbiota, Pathophysiology

## Abstract

The gut microbiota has been associated with many diseases, including endometriosis. However, very few studies have been conducted on this topic in human. This study aimed to investigate the association between endometriosis and gut microbiota. Women with endometriosis (*N*=66) were identified at the Department of Gynaecology and each patient was matched with three controls (*N*=198) from the general population. All participants answered questionnaires about socioeconomic data, medical history, and gastrointestinal symptoms and passed stool samples. Gut bacteria were analyzed using 16S ribosomal RNA sequencing, and in total, 58 bacteria were observed at genus level in both patients with endometriosis and controls. Comparisons of the microbiota between patients and controls and within the endometriosis cohort were performed. Both alpha and beta diversities were higher in controls than in patients. With the false discovery rate q<0.05, abundance of 12 bacteria belonging to the classes Bacilli, Bacteroidia, Clostridia, Coriobacteriia, and Gammaproteobacter differed significantly between patients and controls. Differences observed between patients with or without isolated ovarian endometriosis, involvement of the gastrointestinal tract, gastrointestinal symptoms, or hormonal treatment disappeared after calculation with false discovery rate. These findings indicate that the gut microbiota may be altered in endometriosis patients.

**Supplementary Information:**

The online version contains supplementary material available at 10.1007/s43032-021-00506-5.

## Introduction

Endometriosis is an inflammatory, estrogen-dependent disease defined by presence of endometrial tissue outside the uterine cavity, affecting approximately 6–10% of reproductive women [1, 2]. Besides gynecological symptoms [3, 4], gastrointestinal symptoms affect up to 90% of patients with endometriosis [5]. The most common gastrointestinal symptom is bloating, followed by nausea, constipation, diarrhea, and vomiting [5, 6].

The gastrointestinal tract is a complex ecosystem with a symbiosis of food molecules, gut mucosal cells, immune system cells, and microorganisms. It is a dynamic environment, and the commensal bacteria, or microbiota, are contently changing [7]. The gut microbiota is thought to play a major role in the maintenance of health and development of disease and has through inflammatory and metabolic changes been proven to affect conditions both inside and outside of the gastrointestinal tract [8–10].

Systemic levels of estrogen in post-menopausal women have been associated with fecal microbiome richness and levels of fecal Clostridia taxa [11]. Therefore, the gut microbiota has been suggested to have an impact on estrogen levels in men and post-menopausal women and to be involved in estrogen-dependent diseases [11, 12]. Higher estrogen levels stimulate epithelial proliferation in the female reproductive tract and have been shown to drive diseases such as endometriosis and endometrial cancer [13]. Recent studies have shown that the gut microbiota is a major regulator of inflammatory processes outside the gastrointestinal tract [14], factors which may be involved in the pathogenesis of endometriosis [15]. The cytoplasmic protein AXIN1 is involved in the regulation of apoptosis and has been reported to be a potential new biomarker for endometriosis [16], with correlations to clinical data such as gastrointestinal symptoms and hormone treatment [17].

Due to the impact of immunological and hormonal changes in patients with endometriosis, and the impact of gut microbiota on immune and estrogen responses, it has been hypothesized that the gut microbiota is involved in the pathogenesis of endometriosis [18]. The primary aim of the present study was to investigate the gut microbiota in endometriosis in comparison with healthy controls. The secondary aim was to examine any differences regarding microbiota abundance within the endometriosis cohort, dependent on disease localization, symptoms, or treatment.

## Materials and Methods

### Study Design

Patients with endometriosis (*N*=66) were recruited from the Department of Gynaecology at Skåne University Hospital and were matched with three controls each from the Malmö Offspring Study (MOS). The study participants answered a questionnaire concerning sociodemographic data and medical history, completed the visual analog scale for irritable bowel syndrome (VAS-IBS), and passed stool samples. The gut microbiota was identified at genus level using 16S rRNA sequencing. Comparisons in gut microbiota were performed between patients and controls, and within the patient cohort, using two-tailed Mann-Whitney *U* test, Fisher’s exact test, Spearman’s correlations test, Shannon diversity index, and Bray Curtis dissimilarity index.

### Patients

Patients were recruited from the Department of Gynaecology at Skåne University Hospital, Malmö, according to the ICD-10 classification of endometriosis, N80. The main inclusion criteria were to have a diagnosis of endometriosis, confirmed by laparoscopy or laparotomy. General inclusion criteria were an age above 18 years and to comprehend the Swedish or English languages. Exclusion criteria were an uncertain diagnosis of endometriosis, current pregnancy, diagnosed inflammatory bowel disease (IBD), living far from the hospital, and multiple or severe somatic or psychiatric comorbidities. Between September 2016 and March 2017, 266 women who fulfilled the inclusion criteria were identified. Of these, 196 women were excluded because they were as follows: unwilling to participate (*N*=162), had moved too far from the hospital (*N*=23), had a non-surgically confirmed diagnosis (*N*=7), or an uncertain diagnosis of endometriosis (*N*=2). This reduced the number of women to 72. Out of these, 66 women passed stool samples and were thereby included in the present study.

### Controls

The controls were recruited from 2644 individuals who had previously been extracted from the MOS [19], which consists of descendants to participants in the Malmö Diet and Cancer Cardiovascular Cohort (MDC-CC). The recruitment of participants to the MDC-CC and the MOS took place in the 1990s and 2010s, respectively [20, 21]. Each case was matched with three controls according to sex (only women), age (± 730 days), body mass index (BMI) (± 2 BMI units), and smoking. The participants from MOS who had not answered questionnaires and passed stool samples or were diagnosed with celiac disease, Crohn’s disease, ulcerative colitis, irritable bowel syndrome (IBS), or lactose intolerance were excluded from the matching process.

### Study Questionnaires

The patients with endometriosis answered questions regarding their endometriosis-associated symptoms such as onset of symptoms, trigger factors, and treatment. They also answered a questionnaire regarding education, occupation, marital status, smoking habits, alcohol habits, physical activity, medical history, and pharmacological treatments. The participants in the MOS answered a similar questionnaire. They were also asked the following question: “Have you experienced bowel symptoms during the last 2 weeks?” All participants from MOS who had answered “yes” were excluded from the present study.

### VAS-IBS

The VAS-IBS is a psychometrically validated questionnaire used to estimate gastrointestinal symptoms in patients with functional bowel disease [22]. With the VAS-IBS questionnaire, the patients estimated the severity of symptoms over the last 2 weeks regarding abdominal pain; constipation; diarrhea; bloating and flatulence; vomiting and nausea; and intestinal symptom’s influence on daily life. The VAS-IBS questionnaire measures each of the symptoms on a continuous scale from 0 to 100 mm where 0 represents no problems and 100 represents very severe problems. The scales were inverted from the original scales [22]. Reference values from 52 healthy women were used as controls [23].

### Gut Microbiota and Laboratory Analyses

Stool samples were collected by the patients and controls at home in sterile tubes (Sarstedt, Numbrecht, Germany) and put in the freezer until they were brought to the lab. The samples were kept at –80 °C until the extraction of microbial DNA.

Microbial DNA was extracted from the stool samples using a QIAamp column Stool Kit. The V1–V3 regions of the 16S ribosomal RNA gene were pairwise (300*2 base pairs) amplified and sequenced using a HiSeq Illumina at GATC Biotech (Constance, Germany). The sequences were stored as fastq files which were aligned by FLASH and binned together to operational taxonomic units (OTUs) using QIIME [24, 25]. The sequences were then matched with the reference database Greengenes and classified at genus level. In total, 937,892,146 reads, with an average of 434,008 reads per sample, were included in the analysis. Finally, the data was normalized using the cumulative sum scaling with the R package *metagenomSeq*.

In total, 64 bacteria at genus level occurred in the control group and 66 occurred in the group of patients with endometriosis. Bacteria that only occurred in <10 of the samples were excluded, leaving 58 bacteria at genus level in the control group and 62 in the patient group, and thus, 58 bacteria were included in the statistical analyses between patients and controls, and 62 bacteria in calculations within the endometriosis cohort.

Levels of plasma AXIN1 and fecal calprotectin were analyzed by ELISA as previously described by Dihm et al. [17].

### Data Categorization

Smoking habits were divided into current smoking and no current smoking, regardless of previous smoking habits. Alcohol intake was divided into <1 or ≥1 standard glass per week. Physical activity was divided into <1 or ≥1 h per week of activity which lead to breathlessness. Hormone treatment was divided into current treatment or no current treatment, regardless of previous treatment. Hormonal treatment included estrogen, combined oral contraceptives, progestin, and gonadotropin-releasing hormone (GnRH) analogs. Localization of endometriosis lesions were divided into isolated ovarian lesions or spread to any other location and bowel involvement or no bowel involvement. Gastrointestinal symptoms were divided into having symptoms or not having any symptoms specified on the VAS-IBS scales, i.e., abdominal pain; diarrhea; constipation; bloating and flatulence; vomiting and nausea; and influence of intestinal symptom’s on daily life the last 2 weeks, considered together as one value. If none of the values exceeded a predetermined value determined in a previous study of healthy female volunteers, the patient was categorized as having no symptoms [23].

### Statistical Analyses

Statistical analyses were performed using the software SPSS© statistical computer package version 26 for Windows. Since the distribution of the quantitative data was skewed, descriptive statistics were calculated by the Mann-Whitney *U* test and Spearman’s correlation test. Fisher’s exact test was used for dichotomous variables. A sensitivity analysis was performed where all patients and controls who had received antibiotic treatment in the last 6 months prior to inclusion in the study were removed.

Beta diversity was calculated using the Bray-Curtis dissimilarity index to detect differences in microbiota composition among the groups. Beta diversity was calculated using *vegdist*; further statistical difference for dissimilarity index was tested with *Adonis*, all within the R package *vegan*. Alpha diversity was tested using the Shannon diversity index to analyze diversity of genus among the samples. Alpha diversity was calculated using *diversity*. Furthermore, a variance test (ANOVA) was performed [26].

Values are presented as median and interquartile range or number and percentage. Q-values are the *p*-values adjusted for FDR set at 5% according to the Benjamini-Hochberg method, to adjust for multiple comparisons, and considered our main results [27].

## Results

### Basal Characteristic

A total of 66 women with endometriosis and 198 controls were included in the study, who showed similar basal characteristics (Table 1). The median age of the patients was 37.8 (32.8–43.3) years and the median BMI was 25.0 (22.0–28.0) kg/m^2^. The vast majority of the women had an education from secondary school or university (95.4%) and were either studying or working (83.4%). Most of the patients were non-smokers (84.8%) and drank less than 1 standard glass of alcohol/week (63.6%). Of all, 27 patients (40.9%) had *≥* 1 h/week of physical activity which led to breathlessness. A minority of the patients (28.8%) were living alone. More patients (62.1 vs. 51.5%) than controls (8.1 vs. 17.2%) were currently treated with hormone therapy or analgesic drugs (non-steroidal anti-inflammatory drugs, opioids, paracetamol) (Table 1).

**Table 1:** Basal characteristics in endometriosis patients and controls

Variables	Controls *N*=198	Patients *N*=66	*p*-value
Age, years	37.0 (32.0–44.0)	37.8 (32.8–43.3)	0.88
BMI, kg/m^2^	24.7 (22.1–27.5)	25.0 (22.0–28.0)	0.70
Education level, *N* (%)			1.00
Missing value	2	1	
Graduated primary	8 (4.1)	2 (3.0)	
Graduated secondary	79 (39.9)	27 (40.9)	
Graduated university	109 (55.1)	36 (54.5)	
Occupation, *N* (%)			0.05
Missing value	14	1	
Full time	105 (53.0)	33 (50.0)	
51–99%	49 (24.7)	10 (15.2)	
1–50%	15 (7.6)	7 (10.6)	
Sick or early retirement	3 (1.5)	5 (7.6)	
Unemployed	4 (2.0)	6 (9.1)	
Student	8 (4.0)	5 (7.6)	
Current smoking, *N* (%)	30 (15.2)	10 (15.2)	1.00
Alcohol intake ≥ 1 glass/week, *N* (%)	71 (35.9)	24 (36.4)	1.00
Physical activity ≥ 1 h/week, *N* (%)	93 (47.0)	27 (40.9)	0.48
Lives alone, *N* (%)	44 (22.2)	19 (28.8)	0.18
Hormone treatment, *N* (%)	16 (8.1)	41 (62.1)	<0.001
Missing value	1		
Antibiotic treatment last 6 months, *N* (%)	29 (14.6)	12 (18.2)	0.56
Missing value		1	
Analgesic treatment, *N* (%)	34 (17.2%)	34 (51.5%)	<0.001
Missing value	6		
Visual analog scale for irritable bowel syndrome			
Missing value		1	
Abdominal pain (mm)		47 (13–72)	
Reference values		5 (1–15)	
Constipation (mm)		28 (2–60)	
Reference values		9 (1–22)	
Diarrhea (mm)		17 (2–55)	
Reference values		3 (0–10)	
Bloating and flatulence		62 (20–76)	
Reference values		14 (1–29)	
Vomiting and nausea (mm)		15 (2–50)	
Reference values		2 (0–3)	
Psychological well-being (mm)		37 (13–62)	
Reference values		4 (0–16)	
Intestinal symptoms influence on daily life (mm)		52 (17–80)	
Reference values		2 (0–18)	

Gastrointestinal symptoms were assessed by the visual analog scale for irritable bowel syndrome, 0–100mm, where 0 mm represents no symptoms and 100 mm maximal symptoms [22]. Reference values from healthy controls are shown [23]. Values are presented as median (interquartile range) or numbers (percentage). Mann-Whitney *U* test or Fisher’s exact test. *p*-values <0.05 were considered statistically significant

*BMI* body mass index

### Endometriosis Characteristics

Twenty-seven patients (40.9%) had isolated ovarian endometriosis and 18 patients (27.3%) had involvement of the gastrointestinal tract (Supplementary Table 1). Of the 41 patients who were currently treated with hormone therapy, 20 patients (48.8%) were treated with estrogen or combined oral contraceptives, 19 patients (46.3%) were treated with progestin, and 8 patients (19.5%) were treated with GnRH analogs. The majority of the patients (86.4%) had suffered from gastrointestinal symptoms over the last 2 weeks prior to inclusion in the study. There was no difference in gastrointestinal symptoms between patients with isolated ovarian lesions or spread lesions (*p*=0.71), gastrointestinal tract involvement or not (*p*=1.00), or with or without hormone treatment (*p*=0.47). There was no difference in hormone treatment between patients with isolated ovarian lesions or spread lesions (*p*=0.31) or gastrointestinal tract involvement or not (*p*=0.57). The median value of plasma AXIN1 was 390.0 (357.5–420.0) pg/ml and the median value of feces calprotectin was 25.00 (25.00–29.50) mg/kg. Of the patients, a total of 8 women (12.1%) had been born by caesarean section. There were no significant differences regarding endometriosis characteristics between the subgroups (data not shown).

### Bacterial Analysis

**Fig. 1:**
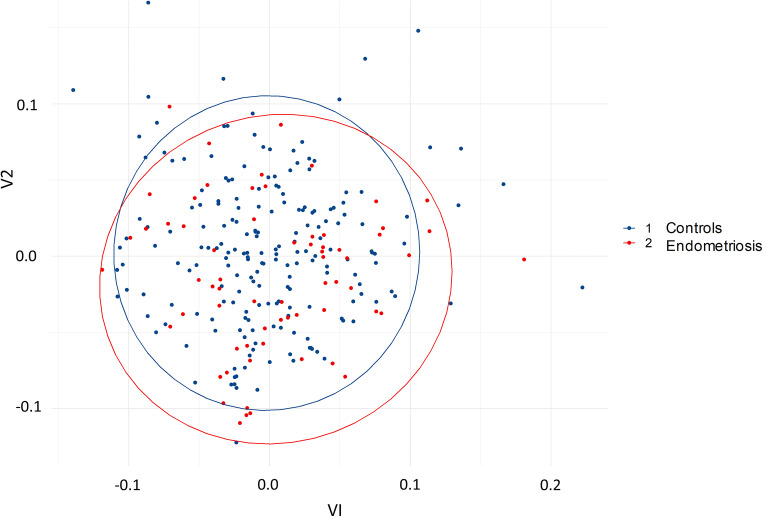
Plot visualizing the beta diversity (Bray-Curtis dissimilarity index) of gut microbiota, colored by healthy controls (1) and patients with endometriosis (2)

**Fig. 2:**
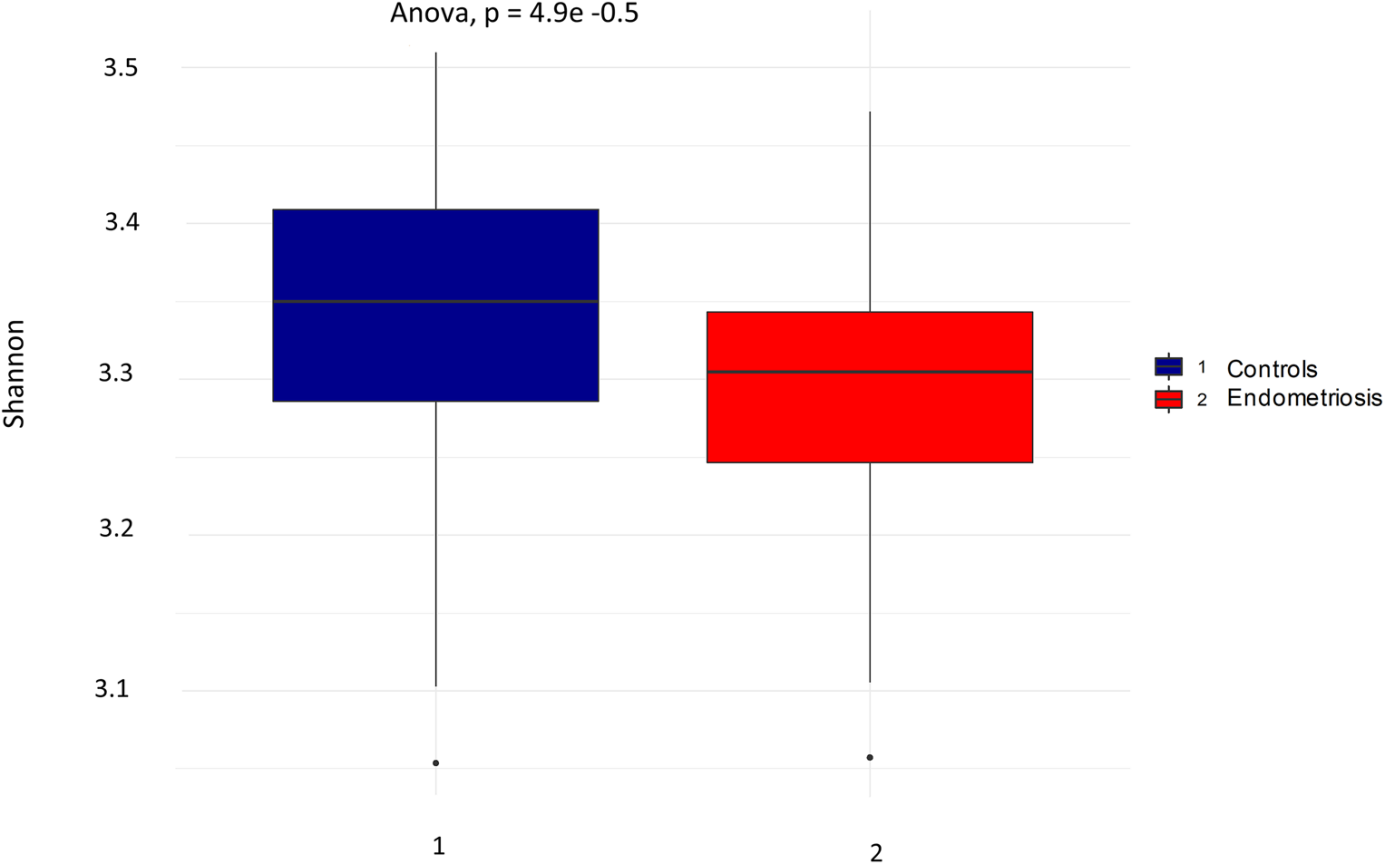
Boxplot of alpha diversity (Shannon diversity index) for gut microbiota in healthy controls (1) and patients with endometriosis (2). *p*-values <0.05 were considered statistically significant

The adonis test showed a significantly higher beta diversity in the control group compared to the endometriosis group. However, *R*^2^ was very small (0.02) (Fig. 1). The ANOVA test showed a significantly higher alpha diversity, *p*=4.9e^−05^, in the control group compared to the endometriosis group (Fig. 2).

**Fig. 3:**
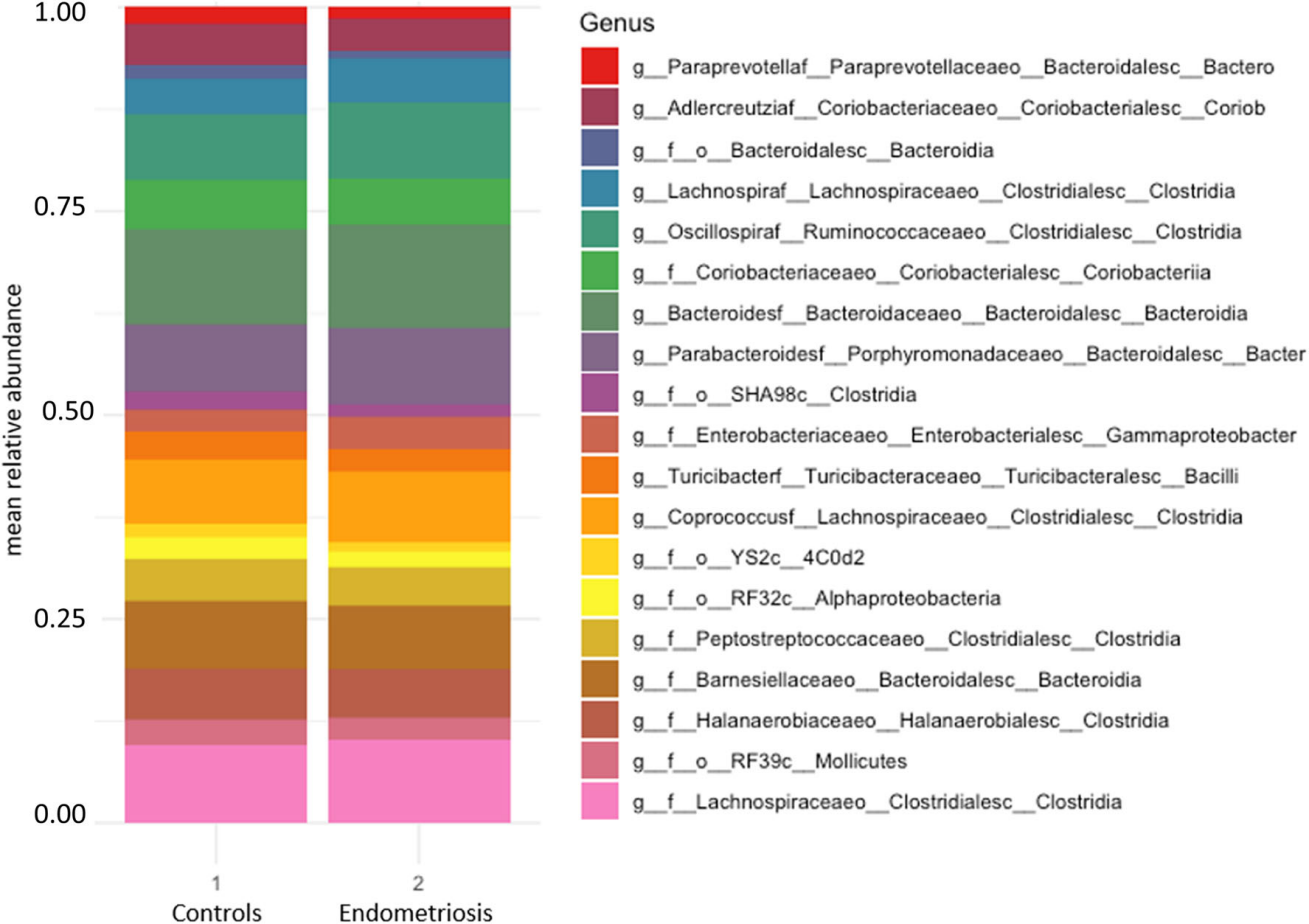
Stacked bar plots of the 19 genus (mean relative abundance) from Table 2 that significantly differed between healthy (group 1) and patients with endometriosis (group 2)

Nineteen gut bacteria at genus level differed in abundance between endometriosis patients and controls. With the FDR set at 0.05, this number was reduced to 12 bacteria (Fig. 3, Table 2). These bacteria belonged to the classes Bacteroidia (*N*=4), Clostridia (*N*=4), Coriobacteriia (*N*=2), Bacilli (*N*=1), and Gammaproteobacter (*N*=1). Two bacteria belonging to the Bacteroidia class (*Bacteroides* and *Parabacteroides*) and two belonging to the Clostridia class (*Oscillospira* and *Coprococccus*) were observed in higher abundance in patients, and two other bacteria in the Bacteroidia (*Paraprevotella *and one unidentified) and Clostridia (*Lachnospira* and one unidentified) classes were observed in lower abundance, compared to controls. Genus belonging to the Bacilli (*Turicibacter*) and the Coriobacteriia (unidentified) classes were found in lower abundance, whereas an unidentified genus in the class of Gammaproteobacter was found in higher abundance, compared to the controls (Fig. 3, Table 2).

**Table 2:** Bacteria with significant difference between endometriosis patients and controls

Bacteria	Controls *N*=198	Patients *N*=66	*p*-value	Q-value
g__Paraprevotella; f__Paraprevotellaceae; o__Bacteroidales; c__Bacteroidia	0.71 (0.00–4.70)	0.00 (0.00–1.11)	<0.001	0.00058
g__Adlercreutzia; f__Coriobacteriaceae; o__Coriobacteriales; c__Coriobacteriia	6.76 (4.91–8.97)	5.15 (3.10–7.31)	<0.001	0.00029
g__f__o__Bacteroidales; c__Bacteroidia	0.63 (0.00–2.69)	0.00 (0.00–0.50)	<0.001	0.00019
g__Lachnospira; f__Lachnospiraceae; o__Clostridiales; c__Clostridia	12.43 (11.60–13.31)	3.47 (1.34–4.88)	<0.001	0.00015
g__Oscillospira; f__Ruminococcaceae; o__Clostridiales; c__Clostridia	10.67 (9.81–11.62)	11.79 (10.60–12.53)	<0.001	0.00012
g__f__Coriobacteriaceae; o__Coriobacteriales; c__Coriobacteriia	8.24 (6.72–9.45)	6.95 (5.25–8.65)	0.001	0.0096
g__Bacteroides; f__Bacteroidaceae; o__Bacteroidales; c__Bacteroidia	15.29 (14.25–16.45)	16.08 (15.14–17.26)	0.001	0.0083
g__Parabacteroides; f__Porphyromonadaceae; o__Bacteroidales; c__Bacteroidia	11.27 (9.98–12.47)	11.92 (10.95–13.20)	0.001	0.0073
g__f__o__SHA98; c__Clostridia	2.63 (0.00–5.70)	0.00 (0.00–4.01)	0.004	0.026
g__f__Enterobacteriaceae; o__Enterobacteriales; c__Gammaproteobacter	3.28 (1.06–5.56)	4.38 (2.30–7.16)	0.007	0.041
g__Turicibacter; f__Turicibacteraceae; o__Turicibacterales; c__Bacilli	4.50 (2.57–6.75)	2.89 (0.00–5.84)	0.008	0.042
g__Coprococcus; f__Lachnospiraceae; o__Clostridiales; c__Clostridia	10.31 (9.34–11.25)	10.81 (9.95–11.76)	0.009	0.044
g__f__o__YS2; c__4C0d2	0.00 (0.00–3.89)	0.00 (0.00–1.11)	0.012	0.054
g__f__o__RF32; c__Alphaproteobacteria	3.27 (0.00–6.72)	0.00 (0.00–5.18)	0.016	0.066
g__f__Peptostreptococcaceae; o__Clostridiales; c__Clostridia	6.90 (5.10–8.60)	6.04 (3.74–8.15)	0.024	0.093
g__f__Barnesiellaceae; o__Bacteroidales; c__Bacteroidia	11.53 (9.43–12.65)	10.43 (7.45–12.55)	0.029	0.11
g__f__Halanaerobiaceae; o__Halanaerobiales; c__Clostridia	8.53 (7.13–9.56)	7.85 (6.12–9.08)	0.033	0.011
g__f__o__RF39; c__Mollicutes	2.86 (0.19–7.70)	0.54 (0.00–6.59)	0.040	0.13
g__f__Lachnospiraceae; o__Clostridiales; c__Clostridia	3.83 (2.37–5.11)	12.72 (12.08–13.57)	0.040	0.12

Values of operational taxonomic unit (OTU) are presented as median (interquartile range). Mann-Whitney *U* test. The q-value is the adjusted *p*-value with a false discovery rate (FDR) of 5% and considered the main result

Patients with isolated ovarian endometriosis had a higher abundance of one unidentified genus and *Lachnobacterium* belonging to the Clostridia class and *Adlercreutzia* belonging to the Coriobacteriia class, compared to those with spread disease (Table 3). Patients with endometrial involvement of the gastrointestinal tract had a higher abundance of *Lactococcus* belonging to the class Bacilli compared to them without involvement (Table 4). Endometriosis patients with gastrointestinal symptoms had a lower abundance of *SMB53* in the Clostridia class, and lower and higher abundance of *Odoribacter* and *Prevotella*, respectively, belonging to the Bacteroidia class, compared to those without symptoms (Table 5). When the abundance of bacteria and the degree of symptoms were compared on bacteria which differed between patients with and without gastrointestinal symptoms, there was a correlation between *Prevotella* and the symptoms constipation (*R*=0.307, *p*=0.014); bloating and flatulence (*R*=0.297, *p*=0.016); and vomiting and nausea (*R*=0.295, *p*=0.017). Patients with hormonal treatment had a higher abundance of *Blautia* and *Ruminococcus* belonging to the Clostridia class, and *Butyricimonas* in the Bacteroidia class, compared with those without treatment (Table 6). There was a correlation between levels of fecal calprotectin and the abundance of *Ruminococcus* (*R*=0.260, *p*=0.038), whereas plasma AXIN1 levels did not correlate with any bacteria abundance (data not shown). With the FDR set at 0.05, these results lost significance, and there were no significant differences of microbiota abundance within the cohort depending on disease localization, symptoms, or hormone treatment (Tables 3, 4, 5, and 6). There was no difference in gut bacteria between patients with and without current analgesic treatment (data not shown).

**Table 3:** Bacteria with significant difference between patients with isolated ovarian and spread endometriosis

Bacteria	Only ovarium *N*=27	Spread *N*=38	*p*-value	Q-value
g__f__Christensenellaceae; o__Clostridiales; c__Clostridia	5.76 (3.86–6.94)	3.77 (0.82–6.10)	0.014	0.868
g__Lachnobacterium; f__Lachnospiraceae; o__Clostridiales; c__Clostridia	6.68 (5.99–7.80)	6.62 (5.21–7.70)	0.036	1.000
g__Adlercreutzia; f__Coriobacteriaceae; o__Coriobacteriales; c__Coriobacteriia	6.30 (4.61–7.30)	4.64 (2.47–6.76)	0.046	0.951

Values of operational taxonomic unit (OTU) are presented as median (interquartile range). Mann-Whitney *U* test. The q-value is the adjusted *p*-value with a false discovery rate (FDR) of 5% and our main results

**Table 4:** Bacteria with significant differences between endometriosis patients with and without involvement of the gastrointestinal (GI) tract

Bacteria	GI tract not involved *N*=47	GI tract involved *N*=18	*p*-value	Q-value
g__Lactococcus; f__Streptococcaceae; o__Lactobacillales; c__Bacilli	2.30 (1.05–4.16)	3.90 (1.81–6.11)	0.034	1.000

Values of operational taxonomic unit (OTU) are presented as median (interquartile range). Mann-Whitney *U* test. The q-value is the adjusted *p*-value with a false discovery rate (FDR) of 5% and our main results

**Table 5:** Bacteria with significant difference between patients with and without gastrointestinal (GI) symptoms

Bacteria	No GI symptoms *N*=8	GI symptoms *N*=57	*p*-value	Q-value
g__SMB53; f__Clostridiaceae; o__Clostridiales; c__Clostridia	6.96 (5.81–8.52)	4.72 (2.54–6.92)	0.011	0.682
g__Odoribacter; f__Odoribacteraceae; o__Bacteroidales; c__Bacteroidia	3.06 (0.54–6.08)	0.00 (0.00–0.82)	0.028	0.868
g__Prevotella; f__Prevotellaceae; o__Bacteroidales; c__Bacteroidia	0.00 (0.00–1.63)	4.96 (2.90–10.44)	0.030	0.620

Values of operational taxonomic unit (OTU) are presented as median (interquartile range). Mann-Whitney *U* test. The q-value is the adjusted *p*-value with a false discovery rate (FDR) of 5% and our main results

**Table 6:** Bacteria with significant difference between patients with and without current hormonal treatment

Bacteria	No treatment *N*=24	Treatment *N*=41	*p*-value	Q-value
g__Blautia; f__Lachnospiraceae; o__Clostridiales; c__Clostridia	10.80 (9.85–12.15)	12.12 (11.05–13.30)	0.009	0.558
g__Ruminococcus; f__Lachnospiraceae; o__Clostridiales; c__Clostridia	8.54 (8.12–10.33)	9.75 (6.82–10.74)	0.019	0.589
g__Butyricimonas; f__Odoribacteraceae; o__Bacteroidales; c__Bacteroidia	8.91 (6.04–10.62)	9.31 (4.27–12.47)	0.034	0.703

Values of operational taxonomic unit (OTU) are presented as median (interquartile range). Mann-Whitney *U* test. The q-value is the adjusted *p*-value with a false discovery rate (FDR) of 5% and our main results

### Sensitivity Analysis

After exclusion of all participants who had received antibiotic treatment in the last 6 months, 17 bacteria differed between the groups in the initial calculation. After FDR adjustment, only three bacteria with a significant difference in abundance between patients and controls were detected, namely* Lachnospira, Oscillospira*, and a genus in the order Bacteroidales (Supplementary Table 2). In patients with sole ovarian involvement, difference in abundance of *Prevotella* was gained whilst those of *Lachnobacterium* and *Adlercreutzia* were lost, compared to those with spread endometriosis (Supplementary Table 3), whereas calculations regarding gastrointestinal involvement were unaffected (Supplementary Table 4). In patients with gastrointestinal symptoms, difference in the abundance of *Turicibacter* was gained whilst those of *Odoribacter *and *Prevotella* were lost, compared to those without gastrointestinal symptoms (Supplementary Table 5). Patients with current hormonal treatment had a difference in abundance of a genus in the family S247, compared to those without treatment, whilst differences of *Ruminococcus* and *Butyricimonas* disappeared (Supplementary Table 6). The differences within the endometriosis cohort lost significance after FDR adjustment (Supplementary Table 3–6).

## Discussion

Generally, the overall diversity of gut microbiota was significantly higher among controls compared to patients with endometriosis. Especially, the alpha diversity differed considerably whilst the beta diversity was only marginally higher in controls than in endometriosis patients. There were differences in abundance of 12 genus belonging to the classes Bacilli, Bacteroidia, Clostridia, Coriobacteriia, and Gammaproteobacter between endometriosis patients and controls, without any significant differences within the endometriosis cohort after FDR adjustments.

A systematic review from 2020 identified 13 clinical studies that investigated the connection between endometriosis and the microbiome [28]. Out of the 13 studies, only six had studied the gut microbiota, and only a single study had been performed in human gut microbiota [28]. The main finding in Ata et al. 2019 [29] was that two women with stage 3–4 endometriosis had an *Escherichia/Shigella-*dominant gut microbiome at genus level, whereas none of the endometriosis-free controls exhibited this dominance. *Escherichia* and *Shigella* belong to the family Enterobacteriaceae. We found a non-significant enrichment of Enterobacteriaceae in endometriosis patients, although we did not identify *Escherichia/Shigella* at genus level using 16 S rRNA sequencing. Since the previous study only included as few as 14 patients [29], and our findings lost significance due to correction for multiple testing, further studies are required to determine the true significance of Enterobacteriaceae between subjects with and without endometriosis. No differences between endometriosis patients and controls in microbiota composition could be found in a recent study examining rectal swab samples [30]. A few studies have examined and found altered microbiota composition in the human reproductive tract in endometriosis, but the low number of studies makes it impossible to estimate the associations between microbiota alterations in the reproductive tract and the gastrointestinal tract [28, 31].

Several animal studies support the hypothesis that endometriosis has an impact on the gut microbiota. Rhesus monkeys with endometriosis had a significantly altered gut microbiota profile compared to healthy controls, and endometriosis was associated with higher concentrations of Gram-negative bacteria and lower concentrations of Lactobacilli [32]. When endometriosis was induced in mice, a higher beta diversity of gut microbiota was developed first after 42 days, with similar alpha diversity, among the endometriosis mice compared with control mice [33]. In contrast, other mouse studies with endometriosis induction have described effects on the gut microbiota already after 21 days; one study found decreased diversity, richness, and abundance of gut microbiota [34] and one found increased alpha and beta diversity [35], whereas a third study could not identify any effect at all [36]. Interestingly, antibiotic treatment to mice reduced the endometriosis lesions and inflammatory responses, changes which were restored after oral feces gavage [35]. Although different models were used to induce and evaluate endometriosis in mice, the results point to an association between endometriosis and gut microbiota and suggest that gut bacteria promote endometriotic lesion progression [31, 34–36]. However, the microbiota alterations may also depend on other functions, e.g., subclinical infections [37]. Our results indicated that the beta diversity was slightly higher in the general population, whilst the alpha diversity was significantly higher, compared to the endometriosis patients. Even though present and previous results are contradicting, which might be explained by different species, our results indicate that long-term exposure to endometriotic tissue may affect the gut microbiota in human.

Endometriosis is an estrogen-dependent disease and high levels of estrogen have been linked to the pathogenesis of endometriosis [13]. The symptoms related to the disease are commonly treated with estrogen, combined oral contraceptives, progestin, or GnRH analogs, which abolish ovulation by lowering of the systemic levels of estrogen [38]. Previous studies have shown that the gut microbiota has an impact on estrogen levels and a bi-directional relationship in estrogen-dependent diseases [11, 12]. Thus, gut microbiota could be involved in the development and symptomology of endometriosis, but endometriosis and its treatment may also affect the composition of gut microbiota. In the present study, we could not prove that changes in gut microbiota would cause or contribute to gastrointestinal symptoms.

A previous study has described an association between the protein AXIN1 and endometriosis in humans [17]. The study showed that plasma levels of AXIN1 were higher in patients with current hormone treatment, positively correlated with both duration and degree of gastrointestinal symptoms, and negatively correlated with levels of fecal calprotectin. However, we could not find any correlation between AXIN1 levels and abundance of bacteria, which differed between those with or without gastrointestinal symptoms or hormonal treatment. The correlation between fecal calprotectin and *Ruminococcus* could possibly reflect more inflammation in the group treated with hormones [17].

The results of the current study did not suggest that the localization of the endometriosis lesions is related to an altered profile of gut microbiota, in accordance with a recent study concerning potential plasma biomarkers for endometriosis, such as AXIN1, ST1A1, CXCL9, and OSM [16]. On the other hand, patients with pelvic endometriosis, with or without ovarian involvement, may have a higher prevalence of tenascin-C autoantibodies than patients with isolated ovarian endometriosis [39].

Antibiotic treatment has been shown to affect the gut microbiota for up to 6 months [40]. In the FDR-controlled sensitivity analysis, only three bacteria at genus level differed significantly between patients and controls. It is unknown whether the reduced number of significant differences in bacteria abundance was due to the lower number of participants in the sensitivity analysis, or if antibiotic treatment had an impact on the results.

The strength of the present study is the examination of a human cohort and matched controls. However, some of the controls could theoretically also suffer from endometriosis. Since all participants from the MOS who suffered from gastrointestinal symptoms were excluded, this risk was minimized. Another limitation is that only 16S rRNA has been examined, and not the whole microbiota genome. Furthermore, adjustments for food and several other confounders were not possible to perform. Examination of biopsy samples instead of feces may be more representative. Also, large numbers of statistical calculations of several bacteria were performed. By using FDR, we have tried to reduce the effect of multiple testing. On the other hand, by using FDR, there is a risk to reduce the significance of a true association, which had been found if a smaller number of bacteria had been measured. Therefore, we have chosen to show also the results before FDR-adjustment.

The large number of different bacteria in the gut in combination with varying lifestyle habits and several confounders for the microbiota composition may lead to different results in different studies, maybe only by chance. One of the great challenges for the future is to standardize sample collection and analysis, with appropriate adjustments for confounders, to be able to compare different studies. Sample collection prior to any endometriosis treatment is important. In addition, associations between the microbiota composition of the gastrointestinal tract and the reproductive tract and estrogen levels would be of interest to evaluate. Furthermore, the microbiota composition should be related to fecal metabolites, to better understand the functional role of the composition [34].

Our results indicate that the overall gut microbial diversity is significantly higher in controls compared to patients with endometriosis. Although the analyses showed significant results for a number of bacteria at the genus level, the differences could be a coincidence depending on multiple comparisons. Based on the cross-sectional study design, it is not possible to decide whether the gut microbiota has any major impact on endometriosis development and the related symptoms or whether endometriosis affects the gut microbiota. However, our study suggests that the gut microbiota may be altered to some extent in patients with endometriosis. The findings put a perspective on microbiota profiling in endometriosis and provides a basis for further research on the pathophysiology, diagnosis, and treatment of endometriosis.

## Supplementary Information

ESM 1 (DOCX 18 kb)

## Data Availability

Data can be provided from the authors upon request.
