# Supplementary material for: Associations Between Endometriosis and Gut Microbiota
Source: Reprod Sci. 2021 Mar 3;28(8):2367–77. doi: 10.1007/s43032-021-00506-5 (PMC8289757; doi:10.1007/s43032-021-00506-5)
Supplement: Supplementary file 1 — (DOCX 18 kb) [file 43032_2021_506_MOESM1_ESM.docx]

# Supplementary Tables

**Table S1.** Localization of endometriosis lesions

| **Localization** | **Number** (%) |
| --- | --- |
| Isolated ovarian | 27 (40.9) |
| Gastrointestinal tract | 18 (27.3) |
| Sacro-uterine ligaments | 9 (13.6) |
| Peritoneum | 8 (12.1) |
| Pouch of Douglas | 7 (10.6) |
| Vesico-uterine pouch | 6 (9.1) |
| Deep infiltrative endometriosis (DIE) | 4 (6.1) |
| Fallopian tubes | 3 (4.5) |

Values are presented as numbers and percentages. With exception of isolated ovarian endometriosis, patients can have lesions on multiple locations.

**Table S2.** Bacteria with significant difference between patients with endometriosis and controls

| **Bacteria** | **Controls**  N=168 | **Patients** N=54 | **P-value** | **Q-value** |
| --- | --- | --- | --- | --- |
| g__f__o__Bacteroidales;c__Bacteroidia | 0.57 (0.00–2.69) | 0.00 (0.00–0.45) | <0.001 | 0.016 |
| g__Lachnospira;f__Lachnospiraceae;o__Clostridiales;  c__Clostridia | 5.61 (4.58–6.50) | 6.65 (5.55–7.71) | <0.001 | 0.016 |
| g__Oscillospira;f__Ruminococcaceae;o__Clostridiales;  c__Clostridia | 10.67  (9.79–11.56) | 11.79  (19.60–12.39) | <0.001 | 0.016 |
| g__f__Coriobacteriaceae;o__Coriobacteriales;c__Coriobacteriia | 8.47 (6.92–9.58) | 7.28 (6.07–8.78) | 0.001 | 0.065 |
| g__Paraprevotella;f__Paraprevotellaceae;o__Bacteroidales;  c__Bacteroidia | 0.71 (0.00–5.30) | 0.00 (0.00–3.09) | 0.002 | 0.082 |
| g__Bacteroides;f__Bacteroidaceae;o__Bacteroidales;  c__Bacteroidia | 12.21  (14.28–16.32) | 15.93  (15.20–17.27) | 0.002 | 0.082 |
| g__Adlercreutzia;f__Coriobacteriaceae;o__Coriobacteriales;  c__Coriobacteriia | 6.64 (4.90–9.07) | 5.37 (3.18–7.57) | 0.004 | 0.115 |
| g__Parabacteroides;f__Porphyromonadaceae;o__Bacteroidales;  c__Bacteroidia | 11.32  (10.03–12.44) | 11.88  (10.79–13.21) | 0.005 | 0.131 |
| g__f__o__RF32;c__Alphaproteobacteria | 3.54 (0.00–6.76) | 0.00 (0.00–4.56) | 0.006 | 0.147 |
| g__Coprococcus;f__Lachnospiraceae;o__Clostridiales;  c__Clostridia | 10.38  (9.33–11.24) | 10.85  (10.17–11.78) | 0.008 | 0.164 |
| g__f__o__YS2;c__4C0d2 | 2.70 (0.00–5.77) | 0.00 (0.00–2.19) | 0.009 | 0.180 |
| g__f__Enterobacteriaceae;o__Enterobacteriales;  c__Gammaproteobacter | 0.14 (0.00–4.12) | 4.16 (2.21–6.50) | 0.011 | 0.197 |
| g__f__o__SHA98;c__Clostridia | 3.05 (1.03–5.38) | 0.00 (0.00–4.56) | 0.015 | 0.123 |
| g__Turicibacter;f__Turicibacteraceae;o__Turicibacterales;  c__Bacilli | 4.46 (2.45–6.75) | 3.21 (0.00–5.84) | 0.034 | 0.230 |
| g__f__Peptostreptococcaceae;o__Clostridiales;c__Clostridia | 6.90 (5.07–8.45) | 5.90 (3.51–8.08) | 0.038 | 0.245 |
| g__Faecalibacterium;f__Ruminococcaceae;o__Clostridiales;  c__Clostrida | 12.31  (5.07–13.63) | 11.80  (10.27–13.29) | 0.047 | 0.262 |
| g__Desulfovibrio;f__Desulfovibrionaceae;o__Desulfovibrionales;  c__Deltaproteobacteria | 0.07 (0.00–4.37) | 0.00 (0.00–2.19) | 0.048 | 0.278 |

All patients and controls who had received antibiotic treatment the last 6 months prior to inclusion in the study were removed from the analysis. Values of operational taxonomic unit (OTU) are presented as median (interquartile range). Mann Whitney U test. The q-value is the adjusted p-value with a false discovery rate (FDR) of 5% and our main results.

**Table S3.** Bacteria with significant difference between patients with isolated ovarian and spread endometriosis

| **Bacteria** | **Only ovarium**  N=24 | **Spread**  N=29 | **P-value** | **Q-value** |
| --- | --- | --- | --- | --- |
| g__f__Christensenellaceae;o__Clostridiales;  c__Clostridia | 5.79  (4.12–7.79) | 3.67  (0.68–6.28) | 0.011 | 0.682 |
| g__Prevotella;f__Prevotellaceae;  o__Bacteroidales;c__Bacteroidia | 0.43  (0.00–1.22) | 0.00  (0.00–0.11) | 0.030 | 0.930 |

All patients and controls who had received antibiotic treatment the last 6 months prior to inclusion in the study were removed from the analysis. Values of operational taxonomic unit (OTU) are presented as median (interquartile range). Mann Whitney U test. The q-value is the adjusted p-value with a false discovery rate (FDR) of 5% and our main results.

| **Bacteria** | **GI tract not involved**  N=38 | **GI tract involved**  N=15 | **P-value** | **Q-value** |
| --- | --- | --- | --- | --- |
| g__Lactococcus;f__Streptococcaceae;  o__Lactobacillales;c__Bacilli | 2.79  (1.01–4.26) | 3.61  (1.91–6.11) | 0.034 | 1.000 |

**Table S4.** Bacteria with significant differences between endometriosis patients with and without involvement of the gastrointestinal (GI) tract

All patients and controls who had received antibiotic treatment the last 6 months prior to inclusion in the study were removed from the analysis. Values of operational taxonomic unit (OTU) are presented as median (interquartile range). Mann Whitney U test. The q-value is the adjusted p-value with a false discovery rate (FDR) of 5% and our main results.

**Table S5.** Bacteria with significant difference between patients with and without gastrointestinal (GI) symptoms

| **Bacteria** | **No GI symptoms**  N=7 | **GI symptoms**  N=44 | **P-value** | **Q-value** |
| --- | --- | --- | --- | --- |
| g__SMB53;f__Clostridiaceae;o__Clostridiales;  c__Clostridia | 7.10  (6.76–8.73) | 4.70  (2.95–6.93) | 0.001 | 0.620 |
| g__Turicibacter;f__Turicibacteraceae;  o__Turicibacterales;c__Bacilli | 6.34  (4.95–7.43) | 2.72  (0.00–5.72) | 0.022 | 0.682 |

All patients and controls who had received antibiotic treatment the last 6 months prior to inclusion in the study were removed from the analysis. Values of operational taxonomic unit (OTU) are presented as median (interquartile range). Mann Whitney U test. The q-value is the adjusted p-value with a false discovery rate (FDR) of 5% and our main results.

**Table S6.** Bacteria with significant difference between patients with and without current hormonal treatment

| **Bacteria** | **No treatment**  N=22 | **Treatment**  N=31 | **P-value** | **Q-value** |
| --- | --- | --- | --- | --- |
| g__Blautia;f__Lachnospiraceae;o__Clostridiale;c__Clostridia | 10.59  (9.78–12.30) | 12.17  (11.11–13.36) | 0.006 | 0.372 |
| g__f__S247;o__Bacteroidales;c__Bacteroidia | 3.76  (0.78–8.24) | 1.25  (0.44–3.31) | 0.039 | 1.000 |

All patients and controls who had received antibiotic treatment the last 6 months prior to inclusion in the study were removed from the analysis. Values of operational taxonomic unit (OTU) are presented as median (interquartile range). Mann Whitney U test. The q-value is the adjusted p-value with a false discovery rate (FDR) of 5% and our main results.
